# Modifier Genes as Therapeutics: The Nuclear Hormone Receptor Rev Erb Alpha (*Nr1d1*) Rescues *Nr2e3* Associated Retinal Disease

**DOI:** 10.1371/journal.pone.0087942

**Published:** 2014-01-31

**Authors:** Nelly M. Cruz, Yang Yuan, Barrett D. Leehy, Rinku Baid, Uday Kompella, Margaret M. DeAngelis, Pascal Escher, Neena B. Haider

**Affiliations:** 1 Schepens Eye Research Institute, Massachusetts Eye and Ear Infirmary, Boston, Massachusetts, United States of America; 2 Department of Ophthalmology, Harvard Medical School, Boston, Massachusetts, United States of America; 3 Department of Genetics, Cell Biology, and Anatomy, University of Nebraska Medical Center, Omaha, Nebraska, United States of America; 4 Nanomedicine and Drug Delivery Laboratory, Department of Pharmaceutical Sciences, University of Colorado Anschutz Medical Campus, Aurora, Colorado, United States of America; 5 Department of Ophthalmology, University of Colorado Anschutz Medical Campus, Aurora, Colorado, United States of America; 6 Ophthalmology and Visual Sciences, John A. Moran Eye Center, Center for Translational Medicine, University of Utah, Salt Lake City, Utah, United States of America; 7 Institute for Research in Ophthalmology, Grand-Champsec Sion, Switzerland; University of Cologne, Germany

## Abstract

Nuclear hormone receptors play a major role in many important biological processes. Most nuclear hormone receptors are ubiquitously expressed and regulate processes such as metabolism, circadian function, and development. They function in these processes to maintain homeostasis through modulation of transcriptional gene networks. In this study we evaluate the effectiveness of a nuclear hormone receptor gene to modulate retinal degeneration and restore the integrity of the retina. Currently, there are no effective treatment options for retinal degenerative diseases leading to progressive and irreversible blindness. In this study we demonstrate that the nuclear hormone receptor gene *Nr1d1* (*Rev-Erbα*) rescues *Nr2e3-*associated retinal degeneration in the *rd7* mouse, which lacks a functional *Nr2e3* gene. Mutations in human *NR2E3* are associated with several retinal degenerations including enhanced S cone syndrome and retinitis pigmentosa. The *rd7* mouse, lacking *Nr2e3*, exhibits an increase in S cones and slow, progressive retinal degeneration. A traditional genetic mapping approach previously identified candidate modifier loci. Here, we demonstrate that *in vivo* delivery of the candidate modifier gene, *Nr1d1* rescues *Nr2e3* associated retinal degeneration. We observed clinical, histological, functional, and molecular restoration of the *rd7* retina. Furthermore, we demonstrate that the mechanism of rescue at the molecular and functional level is through the re-regulation of key genes within the *Nr2e3*-directed transcriptional network. Together, these findings reveal the potency of nuclear receptors as modulators of disease and specifically of NR1D1 as a novel therapeutic for retinal degenerations.

## Introduction

Genetic heterogeneity (identical mutations with variable clinical presentation of disease) is a common feature for many Mendelian disorders [1]. While the underlying mechanisms causing such variations are case specific, it is clear that environmental factors, allelic heterogeneity, genetic modifiers, or a combination of these, can have a profound impact in disease expressivity. Genetic modifiers are allelic variants distinct from the disease-causing gene that can alter disease onset, progression or the clinical features for that particular disease [2]. Since genetic modifiers can result in either increasing or reducing disease severity, identification of modifier loci is important for understanding disease pathophysiology, predicting disease progression and developing novel therapeutic strategies. Modifier loci have been mapped for several diseases in both human and mouse, including cystic fibrosis, epilepsy, Huntington’s disease, hearing loss and retinal degeneration [3]–[14]. The availability of extensive genomic tools and multiple inbred strains of mouse models provide a unique platform to uncover genetic modifiers that strongly influence phenotypic variation in human disease [15]. As such, identification of these modifier genes also provides powerful and novel therapeutics.

While significant disease variability is observed for inherited retinal degenerative diseases, the underlying causes for such variations are largely undiscovered [13], [16]–[19]. Mutations in the nuclear hormone receptor NR2E3, also known as photoreceptor-specific nuclear receptor (PNR), have been associated with several retinal diseases including enhanced S-cone syndrome (ESCS), Goldmann-Favre syndrome and retinitis pigmentosa [20]–[25]. NR2E3 functions as dual activator and suppressor of gene expression and, together with transcription factors such as NRL, CRX and NR1D1, modulates photoreceptor cell fate and differentiation [26]–[31]. NR2E3 is also expressed in mature photoreceptors where it regulates expression of genes essential for proper function, such as phototransduction genes [31], [32]. The NR2E3 protein contains four evolutionary conserved domains that are shared by the nuclear hormone receptor family; namely the highly variable A/B domain, N terminal DNA binding domain, a flexible hinge region and the ligand-binding and dimerization domain in the C terminus [33], [34]. More than 30 disease-causing mutations have been identified in *NR2E3*, most of which are located within the DNA binding domain and the ligand-binding domain [20]–[25]. While most *NR2E3* mutations have a recessive mode of inheritance, a c.166G>A (p.G56R) mutation in the *NR2E3* gene is associated with autosomal dominant retinitis pigmentosa [25]. These data show significant phenotypic variation in patients with *NR2E3* associated retinal degeneration. Interestingly, variable clinical presentation is observed even in patients harboring the same mutation and within the same family, suggesting that modifier genes modulate disease outcome in these patients [21], [35], [36].

The retinal degeneration 7 (*rd7*) mouse is a model for *Nr2e3* associated retinal disease [37], [38]. We utilize the *Nr2e3^rd7/rd7^* mouse model to study the genetic heterogeneity observed in *Nr2e3* associated retinal degeneration and to identify genetic modifiers that contribute to such variation. Mice homozygous for the *rd7* mutation develop retinal dysplasia, with whorls and rosettes apparent at postnatal day 10 (P10) and retinal spots detectable by fundus examination at eye opening (P14) [37]–[39]. Similar to patients with *Nr2e3* mutations, *rd7* mice exhibit significant increase of S-cones and progressive degeneration of rod and cone photoreceptor cells [38]. Our previous studies demonstrated that the *rd7* phenotype is highly variable depending on genetic background [40]. We observe complete penetrance in the B6.Cg-*Nr2e3^rd7/rd7^* strain, while suppression occurs in crosses with the genetically divergent and inbred strains AKR/J, CAST/EiJ and NOD.NOH-H2^nb1^, revealing that modifier alleles are conferring resistance or susceptibility to the *Nr2e3^rd7/rd7^* phenotypes [40].In this study, we identified the nuclear hormone receptor *Rev-erb alpha*, hereafter referred to as *Nr1d1*, as a genetic modifier of *Nr2e3^rd7/rd7^*. We genetically fine mapped a locus on chromosome 11 linked to *Nr2e3^rd7/rd7^* suppression in the AKR/J background and through sequence analysis, identified two strain-specific variations in the *Nr1d1* gene within this locus. Delivery of the *Nr1d1* gene to the retinas of B6.Cg-Nr2e3*^rd7/rd7^* neonates rescues retinal spotting and retinal dysplasia associated with *Nr2e3* loss, confirming that increased *Nr1d1* expression is sufficient for suppressing *rd7*. Importantly, we show that *Nr1d1* delivery results in re-regulation of key genes within the *Nr2e3*-directed network that are essential for proper photoreceptor function. Our findings uncover NR1D1 as a potential therapeutic target for *Nr2e3* associated retinal degeneration that can compensate for *Nr2e3* loss by regulating key molecular pathways associated with disease.

## Results

### Genetic Fine Mapping of *rd7* Modifier Locus on AKR/J Chromosome 11

**Figure 1:**
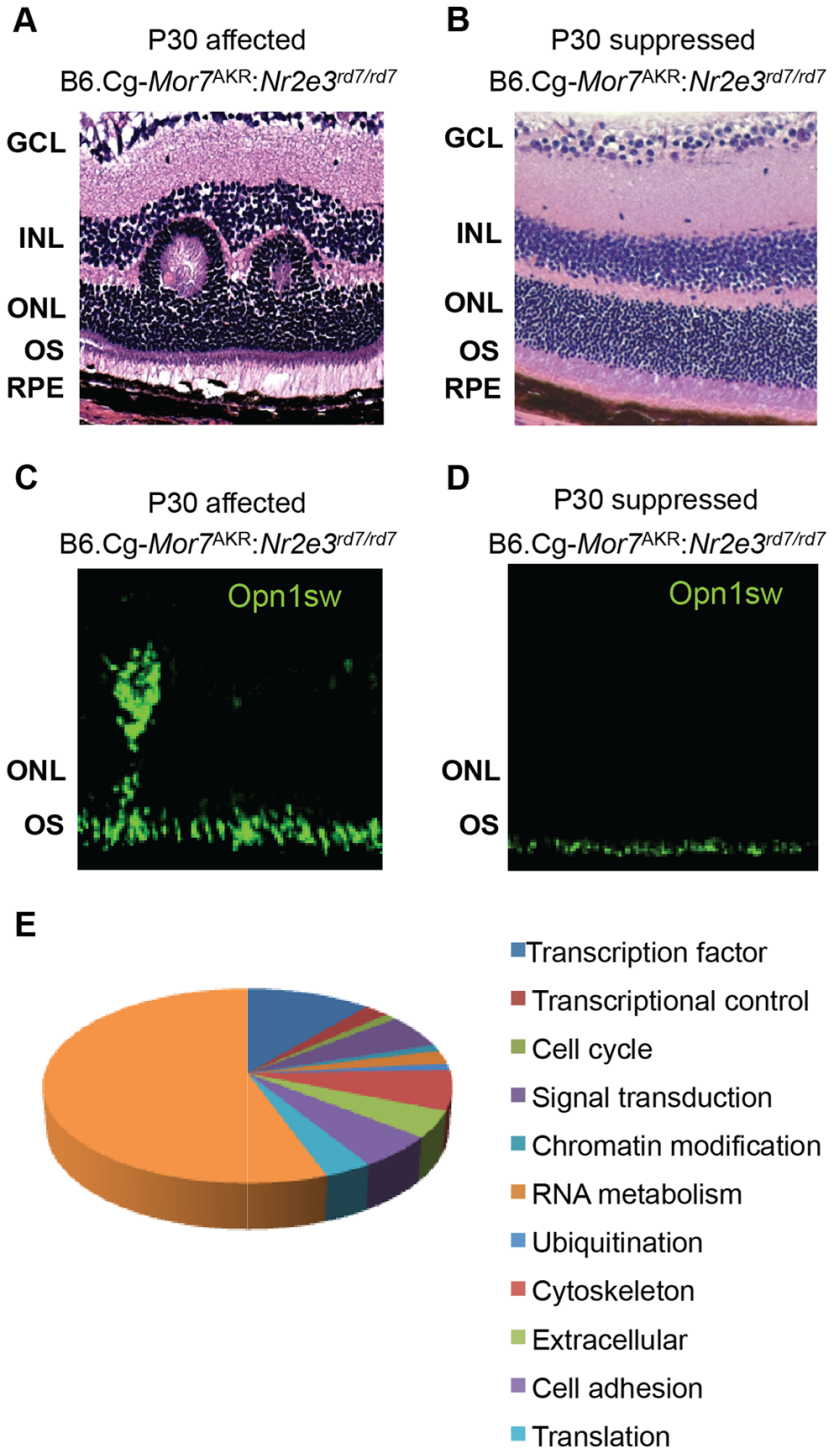
*rd7* phenotypes are suppressed in N6 B6.Cg-*Mor7* ^AKR^:*Nr2e3^rd7/rd7^* mice. (A, B) Hematoxylin and eosin staining of retinal sections from affected (A) and suppressed (B) F_2_ B6.Cg-*Mor7* ^AKR^:*Nr2e3^rd7/rd7^* P30 animals. Retinal dysplasia was absent in the suppressed *rd7* homozygote animals. (C, D) Labeling of retinal sections with anti*-*OPN1SW shows that the S-cone population is restored to a normal level in suppressed F_2_ Cg.AKR/J-*Nr2e3^rd7/rd7^* animals (D), compared to affected animals (C). (E) Chart showing distribution of the 95 retinal genes that map to the *Mor7* interval. GCL: ganglion cell layer, INL: inner nuclear layer, ONL: outer nuclear layer, OS: outer segments, RPE: retinal pigment epithelium.

Our previous studies revealed that genetic background strongly influences penetrance of *Nr2e3^rd7/rd7^* phenotypes [37], [40]. Specifically, complete suppression of *rd7* retinal degeneration was observed in outcrosses of B6.Cg-*Nr2e3^rd7/rd7^* mice to AKR/J, CAST/EiJ or NOD.NOH-H2^nb1^ mice and several modifier loci that were unique for each strain were identified [40]. Two suggestive quantitative trait loci (QTL) located on chromosomes 7 and 11 were found to be associated with suppression in the AKR/J genetic background [40]. To determine if a single modifier gene is able to ameliorate *rd7* associated retinal degeneration, we generated an incipient congenic strain that harbors the AKR/J modifier locus on chromosome 11, named *Mor7* for modifier of *rd7,* by backcrossing F_2_ progeny from our B6.Cg-*Nr2e3^rd7/rd7^* × AKR/J cross to the C57BL/6J inbred strain for six consecutive generations. Congenic animals carry the modifier loci from AKR/J on a C57BL/6J genetic background. Approximately 65% of the B6.Cg-*Mor7* ^AKR^:*Nr2e3^rd7/rd7^* N6 F_2_ animals homozygous for the *rd7* mutation did not exhibited the retinal spotting normally observed in B6.Cg-Nr2e3*^rd7/rd7^* animals, compared to 49% of F_2_ animals from the initial intercross of B6.Cg-Nr2e3*^rd7/rd7^* × AKR/J, suggesting a single modifier gene is sufficient for *rd7* suppression. A genome wide analysis of the congenic F_2_ animals confirmed that approximately 95% of the B6.Cg-*Mor7* ^AKR^:*Nr2e3^rd7/rd7^* genome harbored C57BL/6J alleles in the N6 generation. Two-thirds of the B6.Cg-*Mor7* ^AKR^:*Nr2e3^rd7/rd7^* suppressed mice were heterozygotes across the *Mor7* locus, indicating that the AKR/J *Mor7* allele acts as a dominant protective allele. Consistent with our previous results, the suppressed B6.Cg-*Mor7* ^AKR^:*Nr2e3^rd7/rd7^* mice harboring the modifier allele showed restored retinal morphology (Figures 1A and B) and normal expression of S-cone opsin (*Opn1sw*), compared to affected littermates harboring the susceptible allele (Figures 1C and D). Through our fine mapping analysis, we refined the *Mor7* suppressor locus to a 3.3 cM region in chromosome 11. This region is flanked by markers D11Mit145 and D11Mit360 and contains approximately 200 genes.

### Identification of *Nr1d1* as a Genetic Modifier of *rd7*

**Figure 2:**
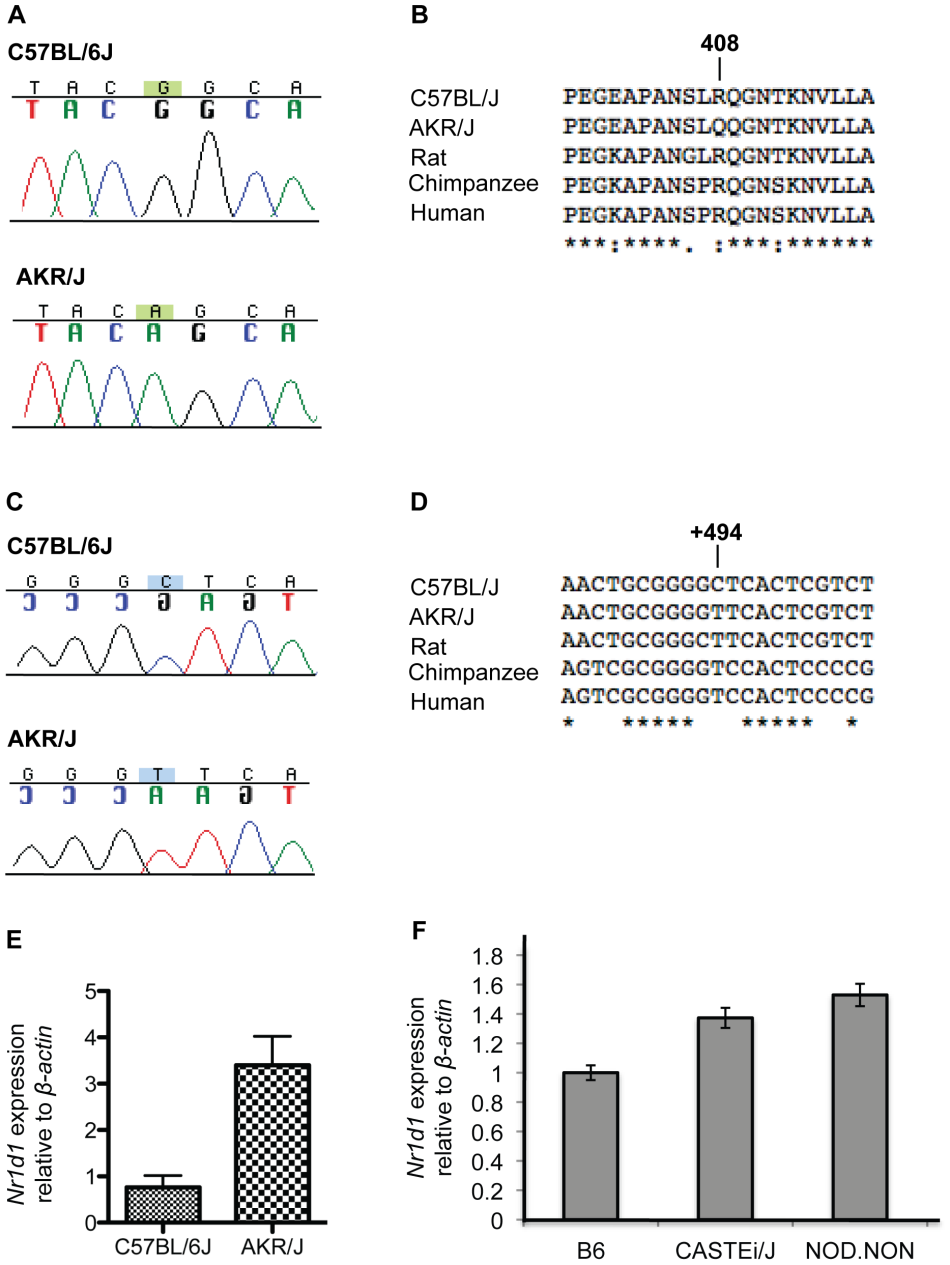
Strain specific alleles and differential expression of *Nr1d1*. (A) C57BL/6J and AKR/J chromatograms of polymorphisms identified in the ligand-binding domain of *Nr1d1.* (B) ClustalW2 sequence alignment of amino acid sequences from C57BL/6J, AKR/J, rat, chimpanzee and human. Stars indicate identity in all sequences, while dots indicate conserved amino acids. (C) C57BL/6J and AKR/J chromatograms of polymorphisms identified in the *Nr1d1* 5′UTR region. (D) ClustalW2 sequence alignment across species reveals the consensus is in accordance with AKR/J sequence. Stars indicate nucleotide conservation in all species. (E) *Nr1d1* relative expression in P30.5 AKR/J and C57BL/6J retinas (mean ± SD of mean, n = 3, p = 0.0024). (F) *Nr1d1* relative expression in P30.5 C57BL/6J, CAST/EiJ and NOD.NOH-H2^nb1^ retinas (p<0.05).

We utilized a candidate approach to identify the *Mor7* gene responsible for conferring *rd7* suppression. Through rigorous *in silico* analysis using several publicly available resources (http://blast.ncbi.nlm.nih.gov, http://www.ensembl.org/index.html, http://pipeline.lbl.gov/cgi-bin/gateway2, http://www.ncbi.nlm.nih.gov/geo/), we determined that 95 of the approximately 200 genes that lie within the *Mor7* locus are expressed in the retina, 10 of which are transcription factors (Figure 1E). We hypothesized that the *Mor7* modifier gene functions in the same or parallel pathway as *Nr2e3*. Three of the identified genes, thyroid hormone receptor alpha (*Thra*), retinoid acid receptor alpha (*Rara*) and rev-erb alpha (*Nr1d1*) are, like *Nr2e3*, members of the nuclear hormone receptor family. Given that several members of this family have been described as key regulators of retinal development and function, *Thra*, *Rara* and *Nr1d1* were considered strong candidates for *Mor7* and their coding as well as upstream regions were sequenced to identify allelic variants between C57BL/6J and AKR/J. While allelic variants were not found in either *Thra* or *Rara*, two single nucleotide polymorphisms (SNPs) were identified in *Nr1d1* at homozygous state (Figure 2).

The SNPs identified in *Nr1d1* are located in both the translated and un-translated regions of the gene. A non-synonymous SNP at position 1222bp was identified in *Nr1d1*, resulting in replacement of the consensus Arginine at position 408 by Glutamine in the AKR/J NR1D1 protein (Figures 2A and B). This SNP is located within the highly conserved ligand-binding domain (LBD) of the NR1D1 nuclear hormone receptor. Specifically, the SNP lies within the binding domain for the NR1D1 co-repressor N-CoR, also known as X domain [41]. A second SNP was identified at position −105 (Figures 2C and D), within the *Nr1d1* promoter region [42], [43]. Specifically, the AKR/J genome harbors a thymidine at this position whereas a cytosine residue is found in C57BL/6J. Sequence alignment of the *Nr1d1* gene across species revealed that T is the evolutionary conserved allele at this position (Figure 2B). As this SNP resides within the putative promoter region of *Nr1d1*, we examined whether *Nr1d1* mRNA expression varies in C57BL/6J versus AKR/J retinas. Quantitative real time PCR confirmed that *Nr1d1* mRNA expression is upregulated by 3 fold in the AKR/J retina, compared to C57BL/6J (P = 0.0024, Figure 2E). This difference in expression may account for the suppressed effect observed in AKR/J genetic background. CAST/EiJ and NOD.NOH-H2^nb1^ genetic backgrounds also confer resistance to *rd7* associated retinal degeneration; however, genetic modifiers on these backgrounds mapped to independent chromosomal locations. [40]. Quantitative real time PCR shows that while *Nr1d1* mRNA levels are increased in CAST/EiJ and NOD.NOH-2^nb1^ wild-type mice compared to B6 (Figure 2F), it is only a slight increase compared to the differences observed for AKR/J vs B6. Thus, the increased levels of *Nr1d1* in CAST/EiJ and NOD.NOH-2^nb1^ may contribute to *rd7* suppression; however, the overall modification of the *rd7* phenotype in those strains is likely due to the presence of other modifier genes.

### *Nr1d1* Delivery Restores Retinal Integrity in *rd7*

NR1D1 regulates many processes such as differentiation, metabolism, and the circadian rhythm [44]. Recently, our studies and those of others have demonstrated a role for NR1D1 in the retina. NR1D1 forms a complex with NR2E3, CRX and NRL, key transcriptional regulators of retinal development and function [26]. Importantly, NR1D1 binds the NR2E3 protein directly and acts synergistically to regulate transcription of photoreceptor-specific genes [26]. Further, our work identified a number of genes co-regulated by NR2E3 and NR1D1 in the developing and adult retina [45]. Thus, *Nr1d1* is a strong candidate to modify the effects of *Nr2e3*-associated retinal degeneration.

**Figure 3:**
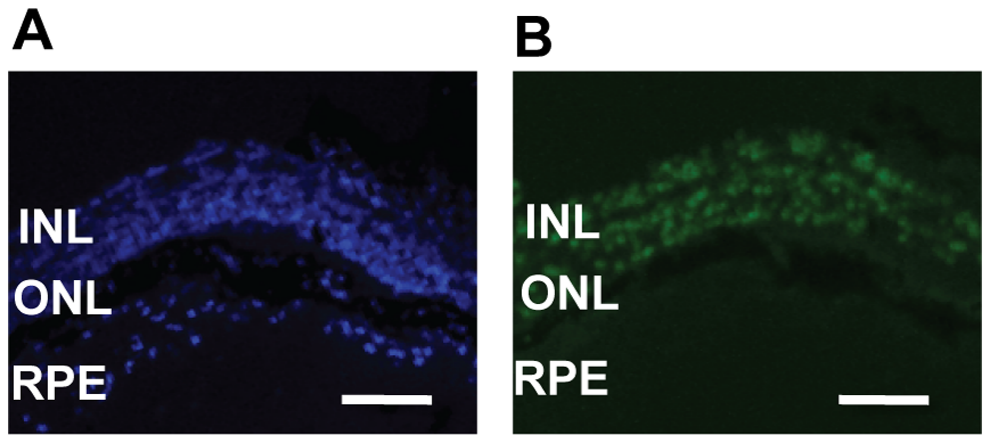
GFP expression in P30 *rd7* retina electroporated with *GFP.Nr1d1^AKR/J^* at P0. (A) GFP expression (green) is apparent in the outer nuclear layer (ONL) and inner nuclear layer (INL) of the retina and co-localizes with the nuclear marker DAPI (blue) (B). INL: inner nuclear layer, ONL: outer nuclear layer, RPE: retinal pigment epithelium. Scale bar = 50 µm.

**Figure 4:**
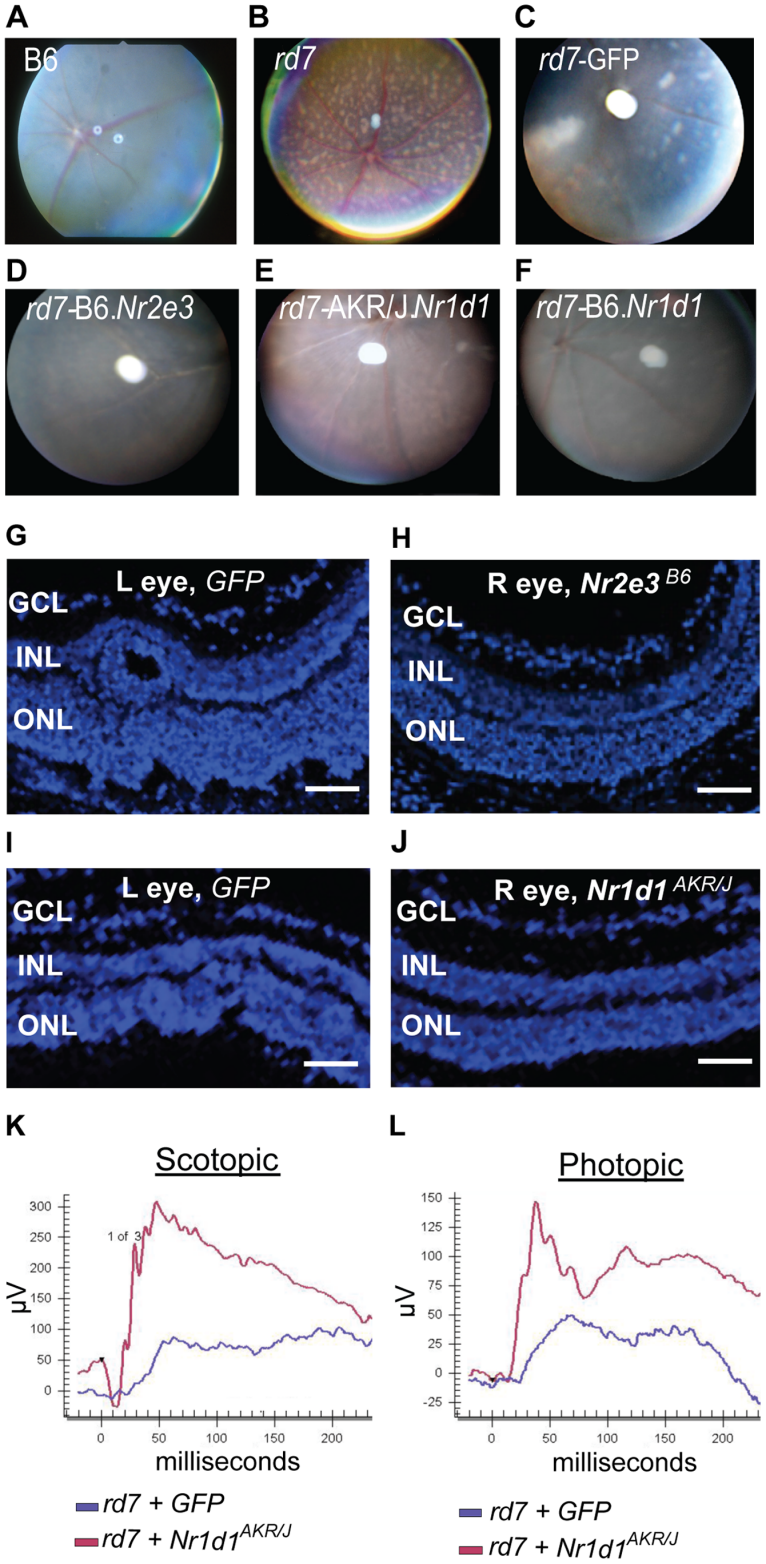
Gene delivery of *Nr1d1* suppresses pan-retinal spotting, retinal dysplasia and function in *Nr2e3* ^rd7/rd7^ mice. (A–F) Fundus photographs of control and *rd7* injected retinas: (A) B6 (uninjected), (B) *rd7* (uninjected), (C) *GFP* injected, (D) *GFP.Nr2e3^B6^* injected, (E) *GFP.Nr1d1^AKR/J^* injected, (F) *GFP*.*Nr1d1* ^B6^ injected. (G–J) DAPI staining (blue) shows rescue of defects in retinal morphology 30 days after electroporation into *rd7* neonatal retinas. (G) GFP control, (H) *Nr2e3^B6^* injected, (I) GFP control, (J) *Nr1d1* ^AKR/J^ injected. L: left, R: right, GCL: ganglion cell layer, INL: inner nuclear layer, ONL: outer nuclear layer. Scale bar = 50 µm. (K, L) Representative scotopic (K) and photopic (L) electroretinograms from animals 4 month after injection with *GFP* (blue) or *GFP.Nr1d1^AKR/J^* (red).

*In vivo* electroporation was performed to deliver *Nr1d1* alleles from either C57BL/6J or AKR/J into the retina of neonatal *rd7* mice and determine whether NR1D1 can modulate *rd7* associated retinal degeneration. The vector used has GFP to detect expression at the site of delivery. One month after delivery, GFP expression was present in both the outer nuclear layer, composed of the cell bodies of rod and cone photoreceptors, and in the inner nuclear layer of the retina (Figure 3). One month after injection, animals were examined clinically by indirect ophthalmoscopy for detection of the characteristic *rd7* pan-retinal spotting. While spotting of the fundus was clearly observable in the eyes electroporated with the control *GFP* vector at P30.5, electroporation of *GFP.Nr2e3* ^B6^ resulted in suppression of the phenotype (Figures 4C and D). Delivery of either *GFP*.*Nr1d1* ^B6^ (B6 allele, without the LBD SNP) or *GFP.Nr1d1* ^AKR/J^ (AKR/J allele, with the LBD SNP) also resulted in rescue of the pan-retinal spotting phenotype (Figures 4E and F). Further, the absence of retinal spotting correlated with absence of retinal dysplasia in histological sections (Figures 4G–J). A subset of the electroporated animals were aged to 4 months and electroretinograms (ERGs) were performed to examine visual function. Significant improvements in both scotopic (dark-adapted) and photopic (light-adapted) ERG response were observed in B6.Cg-*Nr2e3^rd/7rd7^* eyes injected with *GFP.Nr1d1* ^AKR/J^, compared to *GFP* injected eyes (Figures 4K and L). These studies demonstrate that the dosage of *Nr1d1* is sufficient for rescue of *Nr2e3*-associated retinal disease irrespective of the allelic variant in the ligand-binding domain; thus the promoter SNP is likely the protective allele-mediating rescue of disease.

**Figure 5:**
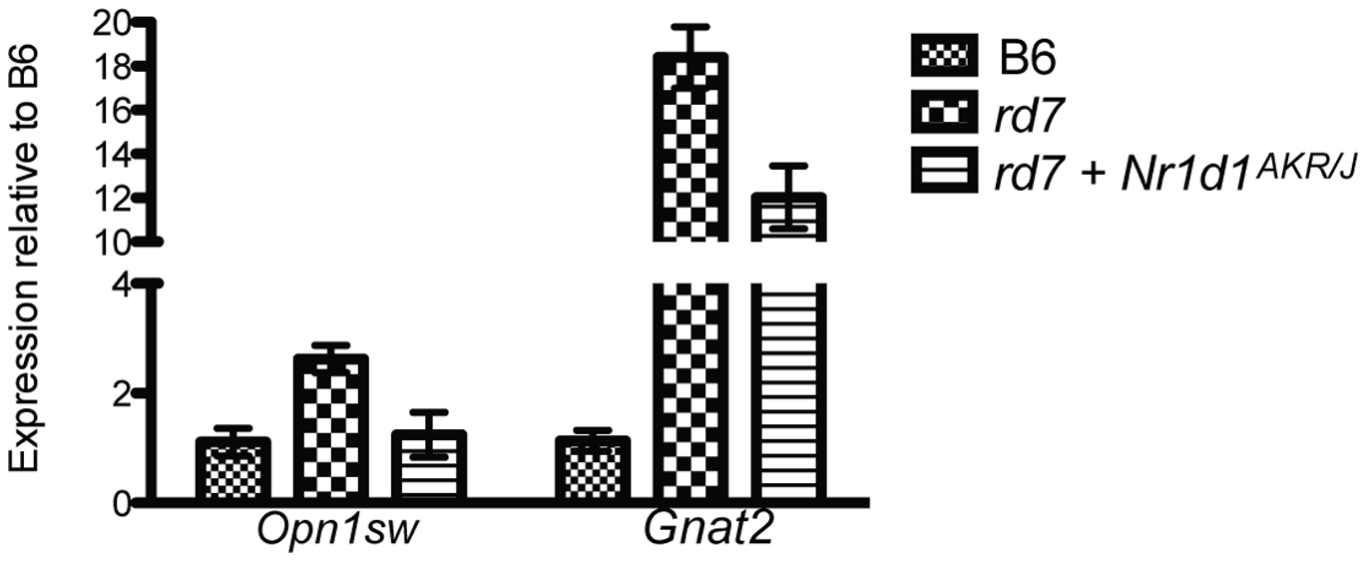
Expression of phototransduction genes *Opn1sw* and *Gnat2* is rescued in *rd7* retinas upon *Nr1d1* delivery. Quantitative real time PCR shows that *Nr1d1* delivery results in down-regulation of the phototransduction genes *Opn1sw* and *Gnat2* in *rd7* retinas (mean ± SD of mean, n = 3, p<0.05), to near normal levels.

***Nr1d1*** **delivery results in the molecular rescue of** ***rd7*** **misregulated genes**NR1D1, a regulator of circadian clock metabolism, also functions as a cofactor of NR2E3 and regulates expression of a number of genes in the retina [26], [45]. We previously characterized the expression profile of the retinas of *rd7* animals and identified 30 genes that are misregulated in *Nr2e3* deficient retinas, 24 of which are directly regulated by NR2E3, NR1D1 or co-regulated by both nuclear hormone receptors [32]. We therefore hypothesized that NR1D1 is able to suppress *rd7* associated retinal degeneration through molecular rescue of key developmental and functional pathways that are misregulated in the *rd7* retina. We specifically focused on evaluating the expression of *Opn1sw* (the S-cone specific opsin) and *Gnat2* (cone photoreceptor specific transducin); key components of the cone phototransduction cascade that are significantly upregulated in *rd7*. We performed quantitative RT-PCR to assay expression of *Opn1sw* and *Gnat2* in the retina of *rd7* animals 30 days after *GFP.Nr1d1^AKR/J^* delivery. Expression of *Opn1sw* was 1.5 fold increased in the retina of untreated *rd7* animals (left eye, n = 3) compared to C57BL/6J, consistent with previous reports (p = 0.004, Figure 5). *GFP.Nr1d1^AKR/J^* delivery to the right eye of the same *rd7* animals resulted in a significant reduction in *Opn1sw* expression (p = 0.035, Figure 5). Furthermore, *Opn1sw* levels in *GFP.Nr1d1^AKR/J^* injected eyes were not significantly different from those present in wild-type C57BL/6J retinas (p = 0.86), indicating that *Nr1d1* delivery rescues *Opn1sw* expression to near normal levels. *Gnat2* was also significantly decreased in eyes injected with *GFP.Nr1d1^AKR/J^*, compared to uninjected eyes from the same animals (p = 0.005, Figure 5). These results suggest that *Nr1d1* up-regulation is able to suppress *Nr2e3* associated retinal degeneration by redirecting the biological networks that modulate photoreceptor development and function.

## Discussion

Photoreceptor biogenesis and homeostasis are directed by key transcription factors that modulate expression of gene networks in an ordered fashion both during development and in the mature retina. Moreover, specific combinations of those transcription factors to regulatory regions are an important mechanism of transcriptional regulation. Several member of the nuclear receptor family, such as NR2E3, regulate key transcriptional networks during these processes. NR1D1 has recently been identified as yet another nuclear receptor important for proper function of the mammalian retina. NR1D1 interacts with NR2E3 and functions in the same transcriptional network [26], [32], [45]. Our previous studies show that acute knockdown of NR1D1 by shRNA targeting in the mouse retina results in retinal degeneration similar to that observed in *rd7* animals [45]. In the present study, we show that *Nr1d1* is a genetic modifier able to ameliorate *Nr2e3* associated retinal degeneration and confirm that NR1D1 and NR2E3 act synergistically to regulate genes involved in retinal development and function.

Delivery of *Nr1d1* alleles from both AKR/J and C57BL/6J strains was able to rescue disease in *rd7*. The data presented here strongly suggests that rescue is mediated by an increase in *Nr1d1* gene dosage and is not dependent on the SNP located in the ligand-binding domain. It is likely that in the AKR/J suppressed animals the promoter SNP results in increased levels of *Nr1d1* that are sufficient to compensate for *Nr2e3* loss. Consistent with this hypothesis, increased *Nr1d1* mRNA expression is observed in the AKR/J retina compared to C57BL/6J. CAST/EiJ and NOD.NOH-H2^nb1^ have slightly higher *Nr1d1* levels compared to C57BL/6J, but the difference is minimal compared to that of AKR/J. We previously showed that outcrossing *rd7* to CAST/EiJ and NOD.NOH-H2^nb1^ leads to suppression of the *rd7* phenotype. Genetic mapping identified unique modifier loci for each strain, distinct from the *Nr1d1-*containing loci that significantly associated with the suppression in ARK/J mice. The minimal increased levels of *Nr1d1* in CAST/EiJ and NOD.NOH-H2^nb1^ thus likely does not influence overall modification of the *rd7* phenotype in those strains, however it may provide some resistance [40].

*Nr1d1* and *Nr2e3* are members of the nuclear hormone receptor family and as such they have a similar gene structure [46]. Further, the NR1D1 and NR2E3 proteins interact with each other directly and regulate common genes/gene networks [26], [45]. This interaction is important for the regulation of gene promoter activation complexes including the proteins NRL and CRX. Therefore, higher *Nr1d1* expression may lead to rescue of *rd7* by increasing active complexes that regulate transcription of genes important for photoreceptor homeostasis and function. This increase in active complexes may lead to an increased activation of low affinity *Nr1d1* response elements that are also *Nr2e3* targets.

As a circadian rhythm regulator, the cellular levels of *Nr1d1* are tightly regulated and are a major determinant of circadian gene expression program [47], [48]. In the retina, both *Nr2e3* and *Nr1d1* mRNA levels oscillate over a 24-hour period [45]. The retina contains an intrinsic circadian clock that regulates many aspects of retinal physiology [49]. More than 2,500 retinal genes have been identified to have circadian expression, including key photoreceptor genes regulated by *Nr2e3* and *Nr1d1* [50]. Therefore, it is likely for changes in *Nr1d1* concentration to result in altered photoreceptor gene expression. We show that delivery of *Nr1d1* to the *rd7* retina re-regulates two key genes required for proper retinal function: *Opn1sw* and *Gnat2*. Both Opn1sw and Gnat2 function in the phototransduction process whereby the retina converts light to an electrical stimulus and as such are essential to vision. We chose to examine these two genes in rescued eyes because they are significantly misregulated in *rd7* animals and are known direct targets of NR2E3. Our data strongly suggest that NR1D1-mediated regulation of key gene networks disrupted by NR2E3 loss contributes to rescue of retinal integrity and function in *rd7* animals.

Our study illustrates that modifier genes capable of modulating a disease state provide viable therapeutic options with broad applicability. We provide evidence that rescue of disease can be achieved by delivering a modifier gene rather than replacing the disease-causing gene. Gene therapy clinical trials have resulted in tremendous success for treating patients with Leber’s congenital amaurosis (LCA), an inherited retinal disease [51]–[55]. These studies demonstrated the safety and efficacy of gene transfer through adeno-associated viral (AAV) delivery and have led to great advancements towards the use of gene therapy in the clinic. We predict that exploiting modifier genes as candidates for gene therapy may significantly broaden the therapeutic potential of AAV to treat retinal diseases and other diseases altered by genetic modifiers.

In summary, this study demonstrates that modifier genes play an integral role in disease presentation and as such can be used as powerful tools for gene therapy that can alter both disease progression and outcome. Future studies will focus on exploring the applicability of using *Nr1d1* gene delivery for treating *Nr2e3*-associated retinal degeneration at advanced stages of disease, as well as retinal disease with other genetic causes. Specifically, this therapeutic approach has a powerful mechanism to treat diseases caused by mutations in different genes that converge on specific nodes or pathways within a common signaling network and as such has a much broader impact than single gene replacement therapy. Further, novel drugs and therapeutics distinct from gene therapy can be developed to exploit the use of genetic modifiers in the clinic.

## Materials and Methods

### Ethics Statement

This study was carried out in strict accordance with the recommendations in the Guide for the Care and Use of Laboratory Animals of the National Institutes of Health. Animal use and procedures were approved by the University of Nebraska Medical Center Animal Care and Use Committee and the Schepens Eye Research Institute Animal Care and Use Committee (Permit Number: S302-0614) in compliance with the Animal Welfare Act Regulations. All efforts were made to minimize animal suffering.

### Animal Maintenance

Animals were housed in vivariums at the Schepens Eye Research Institute and the Nebraska Medical Center. C57BL/6J and AKR/J mice were obtained from Jackson Laboratories, Bar Harbor, ME. B6.Cg-*Nr2e3^rd7/rd7^* has been previously described [40]. B6.Cg-*Mor7* ^AKR^:*Nr2e3^rd7/rd7^* mice were generated by outcrossing B6.Cg-*Nr2e3^rd7/rd7^* × AKR/J F_2_ mice to C57BL/6J, followed by backcrossing of the F_2_ progeny to C57BL/6J for six consecutive generations. Genotyping for the *Nr2e3^rd7/rd7^* mutation was performed as previously described [38].

### Construction of Expression Vectors

cDNA from C57BL/6J or AKR/J mice was used to amplify the *Nr2e3^B6^*, *Nr1d1^B6^ and Nr1d1^AKR/J^* alleles with the following primers: *Nr1d1* (F: TTTTTAAGCTTCATCACAACCTCCAGTTTGT GTC, R: TTTTTAAGCTTGGCGTCCACCCGGAAGGACAGCA) and *Nr2e3* (F: TTTTTAAGCTTGCAAGCAGGCTACCCTTAGGACC, R: TTTTTAAGCTTGAACATGTCA CACAGGAGCTTCT). Amplified sequences spanning the whole coding sequence were cloned into the pAcGFP1-N1 plasmid from Clontech. All plasmids were confirmed by direct sequencing using vector and gene-specific primers.

### *In vivo* Electroporation

*Nr1d1* allele specific constructs (designated as *GFP.Nr1d1* ^B6^ and *GFP.Nr1d1* ^AKR/J^) were delivered by subretinal injection into the right eye of P0.5 *rd7* animals using the electroporation method developed by Matsuda *et al.* [56]. The *Nr2e3* allele from C57B6L/J (*GFP*.*Nr2e3* ^B6^) was electroporated into *rd7* animals as a positive control, while electroporation of empty *GFP* expression vector or no injection to the left eye served as a negative control. 1 µg of naked DNA was injected subretinally, followed by electroporation immediately with tweezer electrodes at five 80 V pulses of 50 ms duration, with 950 ms intervals, using a square wave electroporator.

### Clinical Examination

Animals were examined by indirect ophthalmology at P30 as previously described [40]. Briefly, pupils of animals were dilated with 1% Atropine and a Keeler Vantage indirect ophthalmoscope with a 60-diopter lens was used for fundus examinations.

### Quantitative Real Time PCR

Gene expression analysis was performed using quantitative RT-PCR as previously described [32]. In brief, retinas were dissected rapidly after eye enucleation and placed in Trizol (Life Technologies, Carlsbad, CA) for RNA extraction. Eyes were consistently collected in the early afternoon for each animal in order to eliminate variability due to circadian expression. Two micrograms of total RNA was reverse transcribed using Retroscript (Ambion, Austin, TX). Real-time PCR was performed in technical triplicates with a minimum of three biological replicates using SYBR Green PCR master mix (Applied Biosystems, Warrington, UK). The following primer were used: *Nr1d1* (F: CGGCTCAGCGTCATAATGAA, R: GTTGCCTTGCCGTAGACTGTT); *Opn1sw* (F: ACCTCTAACAATGGGCTGTGTGA, R: GCTGCCGAAGGGTTTACAGA); *Gnat2* (F: CCAGCTGGACCGGATTACAG, R: CAGGTGACTCCCTCGAAGCA) and *β-Actin* (F: ATGCCTCCCCTACCAATCTTC, R: GGATAACGTCCAGGGAACCA). Reactions were quantified using a Roche 480 LightCycler real time PCR instrument. Relative expression levels were normalized to the amount of *β-Actin* expressed and fold change relative to wild-type C57BL/6J control was calculated using the delta Ct method. Standard error was calculated to determine statistical significance.

### Statistical Analysis

Statistical analysis for Figures 2 and 4 was performed using the two-tailed Student’s *t* test, with significance defined as P<0.05. At minimum 3 biological replicates were included in the each experiment.

### Electroretinography

Electroretinogram analysis was performed on 7 mice of each strain (4 month-old), as described previously [40]. Mice were anesthetized with an intraperitoneal injection of a saline carrier (10 mg/g body weight) containing ketamine (1 mg/mL) and xylazine (0.4 mg/mL). Mice were dark adapted for at least six hours and then anesthetized prior to recording. Dark-adapted responses were recorded to short wavelength (λmax = 470 nm; Wratten 47A filter) flashes of light over a 4.0 log unit range of intensities (0.3 log unit steps) up to the maximum allowable by the photic stimulator. Light-adapted responses were obtained with white flashes (0.3 step) on the rod-saturating background after 10 min of exposure to the background light to allow complete light adaptation. Signal processing was performed using EM for Windows v7.1.2. Signals were sampled every 0.8 ms over a response window of 200 ms. Responses were averaged for each stimulus condition with up to 50 records for the weakest signals.
